# Observing the computational concept of abstraction in blind and low vision learners using the Bee-bot and Blue-bot

**DOI:** 10.1080/08993408.2023.2272232

**Published:** 2023-11-16

**Authors:** Anna van der Meulen, Mijke Hartendorp, Wendy Voorn, Felienne Hermans

**Affiliations:** a Leiden Institute of Advanced Computer Science, University of Leiden, Leiden, The Netherlands; b Research Group Employability Transition, Saxion University of Applied Science, Deventer, The Netherlands; c Royal Dutch Visio, Huizen, The Netherlands; d Faculty of Science, Computer Science, Vrije Universiteit Amsterdam, Amsterdam, The Netherlands

**Keywords:** Accessible education, learners with visual impairments, computational practices

## Abstract

**Background and Context:**

In order to fully include learners with visual impairments in early programming education, it is necessary to gain insight into specificities regarding their experience of and approach to abstract computational concepts.

**Objective:**

In this study, we use the model of the layers of abstraction to explore how learners with visual impairments approach the computational concept of abstraction, working with the Bee-bot and Blue-bot.

**Method:**

Six blind and three low vision learners from the elementary school level were observed while completing programming assignments.

**Findings:**

The model of the layers of abstraction, can overall be generalized to learners with visual impairments, who engage in patterns that reflect iterative actions of redesigning and debugging. Especially our blind learners use specific tactile and physical behaviors to engage in these actions.

**Implications:**

Ultimately, understanding such specificities can contribute to inclusive tailored educational instruction and support.

## 1. Introduction

Teaching computational thinking requires insights into how learners understand computational concepts and engage in computational practices. Moreover, to involve all learners and optimize inclusive teaching, it is essential to also know how such understanding and engagement can differ for specific groups of learners, such as those with impairments (Prado et al., 2021). Specifically for the group of learners with visual impairments, attention has risen the past few years to improve their participation in early programming lessons. Most research has focused on usability and accessibility of programming tools and materials. Learners with visual impairments form a challenging group here, because of the wide variety in their vision (with the majority having low vision in diverse forms, and a smaller group being blind) which results in different possibilities and preferences, especially when it comes to the use of technology (Bocconi et al., 2007). General technology accessibility issues are known in for instance screenreader compatibility. Furthermore, materials designed to introduce computational thinking to younger learners are often very visual in nature. Consequently, studies have been striving to identify which specific issues in these materials hinder the accessibility for low vision and blind learners. This has resulted in several proposed and implemented adaptations of materials. Examples are the improvement of accessibility of block-based environments (Koushik et al., 2019; Ludi et al., 2018) and the addition of audio feedback to navigate through textual code (Baker et al., 2015; Stefik et al., 2011). Further, new materials have been specifically designed for the target group, including a tangible block-based tool (Morrison et al., 2018) and inquiries are being made into suited instructions and support (Alotaibi et al., 2020).

Importantly however, the area of specificities in cognitive processing in visually impaired individuals has been rather unexplored. Especially in the subgroup of blind learners it is likely that such specificities exist, due to known particularities in visual-spatial mental modeling and spatial navigation of this group (Thinus-Blanc & Gaunet, 1997; Vecchi et al., 2004). The resulting complexity of conveying abstract cognitive concepts to learners with visual impairments has been documented in other educational fields such as science (Kızılaslan et al., 2019) and music (Antović et al., 2013). In this study, we focus on the field of programming education, and explore how blind and low vision learners approach the computational concept of abstraction. By observing these learners during programming assignments with the educational robots the Bee-bot and Blue-bot, we assess their approach to and experience of this concept through concrete behaviors. This will provide insight into how the process of abstraction emerges in this group of learners. Ultimately, these insights can contribute to understand specificities within their cognitive processing in the context of computational concepts, as well as to provide tailored educational support.

Concerning our language use, we are aware of the discussions on appropriate terms when referring to people with impairments (Sharif et al., 2022). Through this paper, we use the terms currently in place in our educational practice as well as in the academic literature we build on: learners with visual impairments, and blind or low vision learners. As a result, we use the term “braille learner”, following this indication by the schools of blind learners (with possibly some residual vision) who are being taught braille.

### 1.1. The computational concept of abstraction

Computational thinking is a widely applied but complex term in the context of programming education to young learners (Brennan & Resnick, 2012). Several definitions of computational thinking are in use (both in academia and in practice), originating from early papers (Cansu & Cansu, 2019; Papert, 2020; Wing, 2006). In line with these earlier understandings, at its core computational thinking can be understood as a set of problem solving processes. Three aspects can be distinguished: computational concepts, computational practices, and computational perspectives (Cansu & Cansu, 2019; Papert, 2020; Wing, 2006). Computational concepts refer to the content of the processes engaged in while programming, for instance iteration or parallelism. Further, computational practices refer to the activities (which can be cognitive) employed to engage with the concepts, for instance debugging. Finally, less relevant here, computational perspectives involve perspectives designers have of themselves and the world.

A core computational process is abstraction, which involves viewing a situation at various levels of detail, and deciding what details we need and can ignore (Faber et al., 2019; Waite et al., 2018). It can be seen as a form of problem solving, as in the model by Perrenet (Perrenet & Kaasenbrood, 2006) where four layers of abstraction are described to understand how novice learners approach programming tasks. In the original model, these layers were identified in Computer Science education students’ thinking. The layers included the problem layer (the highest layer, where a verbal description of the problem is provided), the design layer (where a detailed depiction of the solution is provided without a reference to the specific programming language), the code layer (referring to the code in the specific programming language) and the execution layer (which involves running the code or referencing to the output, the lowest level). The model has been applied in the context of elementary school-level learners (Faber et al., 2019; Waite et al., 2018), where concrete observable behaviors of young learners while working with an educational robot have been operationalized for each layer (Faber et al., 2019). This enables identifying which layer a learner is engaged in during an assignment. Behaviors include tactile expressions (for instance, pointing towards the robot), verbal expressions (describing the route of the robot) and observing the environment, route, or robot carrying out the task. In addition to observing the behaviors within layers, the model can also be used to assess how learners switch between the layers through pattern analyses (Faber et al., 2019). Previous research with the educational robot revealed that young learners spend little time on the problem layer but do switch between layers in a matter that suggests debugging (switching back to the code layers after the execution) and redesigning (switching back to the design layer).

Level of complexity or abstraction of the problem or task itself can also be taken into account by looking at the dimensions of control and representation (Faber et al., 2019; Kalas et al., 2018). Control can range from direct manipulation (moving a physical robot or dragging a character in a programming environment) to computational control (where a sequence of instructions is constructed that is executed later on). Representation refers to the manner in which such a sequence is presented. This can range from the programmer having no representation to the programmer having an external plan outside of the unit that is being programmed (Faber et al., 2019). More abstract and complex tasks consequently imply moving away from more direct computational control (Kalas et al., 2018).

### 1.2. Abstraction and mental modeling in young blind learners

Understanding and operationalizing abstraction at this observable level is an essential step in optimizing programming education, since it provides direct starting points for teachers to recognize and support the development of this process. It is consequently also key to identify how abstraction emerges in learners with diverse needs. Learners with visual impairments have received quite some attention in the topic of programming education (though young learners more recently) mostly at the level of identifying practical usability and accessibility in programming materials and environments (Hadwen-Bennett et al., 2018; Jašková & Kaliaková, 2014, Kabátová, Jaškoá, Lecký, & Laššáková, 2012; Morrison et al., 2018). Various barriers have been described (Hadwen-Bennett et al., 2018). These include inaccessibility in programming languages and environments and code navigation (Hadwen-Bennett et al., 2018). Specifically for the younger learners, there are relevant issues with block-based languages (including the reliance upon the mouse and the drag and drop interface) (Morrison et al., 2018) as well as the visual properties of tangible materials such as robots and robotic kits (Kabátová et al., 2012; Morrison et al., 2018). At the cognitive level (of comprehending and representing computational concepts and employing particular computational practices) however, specificities in the group of learners with visual impairments have been rather unexplored. We know from other educational fields such as science (Kızılaslan et al., 2019) and music (Antović et al., 2013) that teaching abstract cognitive concepts to learners with visual impairments can be challenging.

This challenge in conveying abstract knowledge to learners with visual impairments can be grounded in specificities in visio-spatial mental modeling and spatial navigation in this group. This is a highly complex area. Generally, qualitative differences can exist for specifically blind compared to sighted individuals in how spatial information is encoded, as a result of the absence of visual experience and the quantitative advantage of vision over other senses (Thinus-Blanc & Gaunet, 1997; Vecchi et al., 2004). However, performance is influenced by several factors, including the onset of blindness, other experiences, taught or developed compensatory mechanisms as well as the specific spatial processing aspects involved or task used. Ultimately, visio-spatial mental images of blind individuals can in practice be functionally equivalent to those of sighted individuals, but differences on specific mental imagery tasks can also be identified. To further understand the implication of this complex picture within the context of young learners with visual impairments’ education, it is recommended to focus on distinct spatial contexts and representations within a particular discipline (DeSutter & Stieff, 2017). Within programming education, the core concept of abstraction provides a suitable starting point. The approach of the layers of abstraction and the previously identified behaviors (Faber et al., 2019) emphasize how more complex tasks and approaches entail less direct manipulation and consequently require more mental modeling. In order to explore this, we use the educational robots Bee-bot and Blue-bot, since these are widely applied in early programming education (Kalas et al., 2018) and have been proven to be at the concrete level relatively accessible for learners with visual impairments (Jašková & Kaliaková, 2014; Kabátová et al., 2012). Further, the two types of this bot provide different options for the dimension of control within a programming task, with the Bee-bot being directly programmed with buttons on the bot and the Blue-bot having the option to be programmed through an external device.

Our research question is: which patterns and specific behaviors, approached from the layers of abstraction model, do children with visual impairments engage in when working on a programming task with the Bee-bot or Blue-bot? Specifically, we will assess how the children move through the abstraction layers during a task, and which behaviors they show within the different layers. In our understanding of the four layers, we follow previous work on this model (Faber et al., 2019; Perrenet & Kaasenbrood, 2006; Waite et al., 2018). Consequently, the problem layer refers to the most abstract level where the problem is discussed, the design layer involves a depiction of the solution, the code layer involves being directly concerned with the code as applicable in the specific tool or language, and the execution is the least abstract layer where the code is ran or where there is preoccupation with the output. In our study, first, frequency of the different layers and switching between the layers can reveal whether learners with visual impairments engage in these higher levels of abstraction, that require mental representation of the problems they work on. Second, how exactly these learners engage in these layers, that is what type of concrete behaviors and practices, are being employed, can indicate what information is used and needed to build these mental representations. This also illuminates the extent to which the original model of the layers of abstraction, based on sighted learners (Faber et al., 2019), is applicable to other learners with certain specificities. Together these insights explore how the cognitive concept of abstraction in the context of programming is experienced by learners with visual impairments. Our primary interest lies in blind children, however given the low prevalence of this group as well as the unexplored nature of this topic in learners with visual impairments overall, we include in our study pairs of learners with visual impairments with each pair containing at least one blind child.

## 2. Methods

### 2.1. Participants

Nine children from three special education schools for learners with visual impairments in the Netherlands participated in pairs in sessions with the Bee-bot (three sessions) and/or the Blue-bot (four sessions). Table 1 summarizes the pairs and characteristics of the children. There were two pairs who participated in both a Bee-bot and Blue-bot session (the pair of sessions 2 and 5 and the pair of sessions 3 and 6). In session 1 and 4 Child 1 was the same, but Child 2 was different.

**Table 1.** Overview of the seven sessions and the participants. */**/*** indicates same pair or same child within pair.

No.	Child1	Child2	Level	Bot
1	Braille (f)*	Braille (m)	Lower	Bee-bot
2**	Low vision (m)	Braille (m)	Lower	Bee-bot
3***	Braille (m)	Braille (m)	Higher	Bee-bot
4	Braille (f)*	Low vision (m)	Lower	Blue-bot
5**	Low vision (m)	Braille (m)	Lower	Blue-bot
6***	Braille (m)	Braille (m)	Higher	Blue-bot
7	Braille (f)	Low vision (f)	Middle	Blue-bot

In total, out of the nine children there were three girls and six boys. Three children had low vision in various forms, including for all of them blurred vision and additional effects such as distorted view or images. The other six children were blind, with two of them being completely blind, and four having some residual vision or light perception. In the results, we refer to the blind learners as “braille learners”, which is how they are indicated at school. This indication reflects that these learners could have some residual vision but were all in any case taught braille, in addition to working with various other tools and assistive technologies such as screenreaders. Because the policy in the Netherlands is that learners with visual impairments enroll in regular education unless not possible, all participants had additional learning issues or other specificities. These included specific behavioral issues or specific learning needs.

The participants were enrolled at the lower, middle or higher level of special education elementary schools. Although there is flexibility in age ranges in this school context, these ranges generally include learners of resp. 6–8, 9–10, and 10–12 years old. The schools were located in different parts of the Netherlands, with the school from pairs 1/4 and 2/5 being in a more rural part and the schools from pair 3/6 and pair 7 in a more urban part. The classes of these schools were similar in size and the schools overall had a similar educational approach and support.

### 2.2. Research design and establishing trustworthiness

The primary focus of this qualitative study was to explore visually impaired learners’ experience of the concept of abstraction in a programming assignment, in order to gain insight into the reality of this topic as experienced by our subjects. Fitting with such a design and focus, we intended to obtain trustworthiness of our study through establishing the four criteria of Guba: trust value, applicability, consistency, neutrality (Guba, 1981; Krefting, 1991). In our collection of the data, we followed a tailored approach fitting the subjects’ specific setting and needs, providing space to find and express their experience. Further, we documented this approach, our sample, and the findings in detailed descriptions (see the relevant sections of the methods and the results sections). As such, we established truth value by staying close and true to the direct experience of subjects and documenting this experience in detail. Further, applicability refers to the extent to which (a type of) generalization is aimed for. In this study, this was limited to enabling transferability to similar participants and contexts by providing details on these participants and contexts. Third, consistency is established in the results section by working with a detailed coding scheme that allows for both pre-defined and newly observed behaviors, and in addition through providing full descriptive pictures for each pair of learners. Finally, neutrality is established again through staying close to our participants’ experience and documenting these experiences in detail.

### 2.3. Procedure

The Bee-bot and Blue-bot sessions were conducted in the context of a larger project on usability and accessibility of programming materials for learners with visual impairments. Classes participating in this project were all part of special education schools part of the two Dutch expertise centres for visual impairments, and three programming lessons focusing on specific materials. The three classes participating in the Bee-bot and Blue-bot lessons had each received the first lesson on an unplugged material, after which they used the Bee-bot in the second lesson and the Blue-bot in the third lesson. Informed consent was obtained from parents, who were approached through the teachers with a letter explaining the lessons and research. Parents were asked to give permission for the participation of their child in the research and for the video recording that took place in the classroom. If a parent would not give consent, their child would still take part in the programming lessons but no data would be collected on this specific child, who would also not be part of the video recording. The latter was ensured by having the children for whom no permission was obtained sit in a separate classroom during the assignment. Half to all of the parents gave consent in the three classes.

The programming lesson consisted of a short introduction, after which the children were divided into pairs to work on an assignment. The introduction was given by the researcher. During the assignment, each pair of children was guided and supported by a tester, this was either the researcher or a research-assistant. The research-assistants were students in social sciences or computer sciences who had received a training on working with children with visual impairments as well as on facilitating the set-up of the assignment as explained below.

### 2.4. Materials and assignment

The educational floor-robot Bee-bot and the more advanced version Blue-bot were both used. These robots have the same basic look and functions, shaped as a bee with a clear front (distinguished by the protruding eyes and nose) and seven buttons on top which can be used to move the bot forward, backwards, turn right or left, pause, and run or erase the program. The type of functions are distinguished by different shapes and colors of the buttons. The bot makes sounds when it is being programmed (with different sounds for a step, for erase, and for run) and when it executes the program (making a sound for each step and a different sound at the end). Whereas the Bee-bot can only be programmed with the buttons on top, the Blue-bot has the option to be programmed externally using the accompanying materials (the tactile reader and tactile reader cards), or the Blue-bot app on a PC or tablet device (the latter was not used in the current study). The tactile reader is an external card holder, which connects to the Blue-bot through Bluetooth. A total number of nine cards, that hold the same five functions as the buttons on top of the robot, can be placed in this holder. Compared to programming the bot with the buttons on top, this external device makes it possible to lay out the program. The original cards indicate their function (step forward, pause, etc.) with a small picture. Since this is unsuited for blind learners, adapted tactile versions of these cards were used (previously created by one of the expertise centres and explored in some of the schools), which contained small tactile shapes attached to the cards below the original pictures. One consideration in the design of the tactile shapes was to find an appropriate alternative for the arrow shape, which had been proved in these previous explorations to be a difficult concept to convey to braille learners. Throughout this study, the tactile versions of the Blue-bot cards were used, as well as (in order to compare) the original versions. Finally, for the environment in which the bots were programmed (see Assignments below), either the wooden board that distinguishes different plates indicating the steps of the Blue-bot and that can be build into a maze was used or loose Kapla blocks to create the environment or a maze.

The sessions with the Bee-bot and Blue-bot started with a plenary introduction to the whole class by the researcher. Since the children had already been introduced to programming during the previous (unplugged) lesson given in the context of the project, this introduction focused on explaining the bot. During this introduction, the children and researcher all sat together, and the bot was passed around all the children for a visual and/or tactile exploration while the researcher explained the buttons (emphasizing the visual, tactile, and auditory elements). In the following Blue-bot sessions, the reader and cards were introduced and passed around all the children. In the lower level class (Sessions 1, 2, 4 and 5) the teacher preferred to conduct most of the explanation individually, after the children had been divided up into pairs.

Once the children had been divided up into pairs and matched to a tester, the tester explained the constructive interaction protocol (described in the next section), after which the video recording was started. Next, the tester checked whether the children had understood the explanation on the bot and if needed provided additional instructions. The assignment always started with the children programming a few steps in order to move the bot from one child to the other. The tester then choose an appropriate assignment to continue, out of several available worked-out assignments with different levels (including having the bot move from point A to point B within an open environment or within a maze, or having the bot perform a dance with a repeated pattern). The children were also allowed to (co-)design the environment and/or think of the end goal for the bot themselves. The children always worked with the bot in a structured manner towards a specific goal. This set-up was designed and carried out following the recommendation for an individually guided, tailored and flexible approach in research with children with impairments (Foss et al., 2013; Guha et al., 2008). In addition to the specific content of the assignment being adapted to the children’s level, tailored extra instruction was provided when required and the teacher was present to intervene for instance when a child got too distracted. Further, in order to gain insights into children’s experience while working with the bots, the think-aloud method of constructive interaction was used (Als et al., 2005). Constructive interaction uses the set-up of a collaboration between children in order to create a natural situation for them to verbalize their experiences (Als et al., 2005). We explicitly stimulated this by providing the children with an elaborate instruction on verbalizing your thoughts at the start, including a concrete example (Als et al., 2005; Donker & Reitsma, 2004; Van Kesteren et al., 2003). The children were instructed to work together and try to verbalize what they were thinking of the material and what they were doing. The tester reminded them throughout the assignment, using neutral prompts (“don’t forget to think aloud”, “what are you doing now”).

### 2.5. Analyses

The sessions were all recorded individually on video. These recordings were processed by coding and transcribing verbal and non-verbal behavior, using a detailed pre-defined coding scheme in line with a theory driven thematic analysis approach (Braun & Clarke, 2006). Continuing on establishing trustworthiness for our study as described in the research design section above, our data analyses were also aimed at capturing the experience of reality of our subjects. This was obtained first of all by staying close to this experience in coding our data, and second by taking into account the learners’ overall approach to the assignment and providing a full picture for each pair of participants on how they proceeded through the assignment. The coding scheme consequently included the primary focus of the study, distinguishing the four layers of abstraction, but also information on additional aspects of the learners’ experience. In addition, the overall impression of the sessions is included in the descriptions in the results as well. The scheme included 17 categories of behaviors referring to specific features of usability and accessibility (for instance-independent use, needed assistance, positive or negative experience) and the computational practices. The behaviors were based on previous insights on the use of programming materials with sighted and visually impaired learners (Donker & Reitsma, 2004; Faber et al., 2019; Milne & Ladner, 2018; Morrison et al., 2018; Read et al., 2002).

Concerning the coding of the computational practices, for each layer, plausible pre-defined behaviors were hypothesized based on previous work with sighted learners (Faber et al., 2019; Perrenet & Kaasenbrood, 2006; Waite et al., 2018) and fitting the Bee-bot and Blue-bot (pre-specified behaviors for each layer can be found in Tables 3–6). In addition, for each layer it was possible to indicate non-anticipated behaviors. This enabled the important intention of the research set-up to explore the current subjects’ behaviors in an open way. For all seven sessions included in this study, a coding scheme was completed, including verbatim transcriptions of verbal behaviors. Behavior occurring during one time or stimulus could be coded within multiple layers, for instance, when children were simultaneously discussing the end point and the steps towards it, which would be coded as pre-defined behavior in the problem as well as design layer. Further, multiple behaviors could also be coded within one layer, when for example children were following the output while discussing whether the outcome was anticipated, which would be coded as two pre-defined behaviors within the output layer. With the detailed and elaborate coding scheme, we aimed to establish consistency in our study, allowing for full descriptive pictures where observed behaviors are embedded in context and connected to the overall experience. Further processing was conducted by first taking an inventory of the frequency of and switching between the layers, by creating frequency tables and a graph for each session representing the switching per layer. We followed the previous paper by Faber et al. (Faber et al., 2019) with this representation through graphs of young learners’ switching through the abstraction layers. Second, the behaviors within the layers were inventoried by creating frequency tables for the pre-specified behaviors within each layer and structuring the open answers for non-anticipated behaviors into patterns. Finally, information from other (not computational practice-related) categories was scanned to obtain an overall picture of the course of the assignment as well as any specificities for each session. Microsoft Excel was used for the coding scheme’s, further processing of the data and creation of the graphs was done in the Statistical Package for the Social Sciences (SPSS), version 27.

**Table 2.** Occurrence of layers within and across sessions. Percentages are relative to specific session.

	1	2	3	4	5	6	7	Total
Problem Layer	24	19	17	5	8	4	5	82
	(8.9%)	(7.3%)	(6.3%)	(3.7%)	(6.5%)	(3.6%)	(7.2%)	(7.0%)
Design Layer	83	106	54	35	21	26	18	343
	(30.6%)	(40.5%)	(19.9%)	(26.1%)	(17.1%)	(23.4%)	(26.1%)	(27.6%)
Code Layer	112	104	73	61	35	18	28	431
	(41.3%)	(39.7%)	(26.9%)	45.5%)	(28.5%)	(16.2%)	(40.6%)	(34.7%)
Execution layer	52	33	127	33	59	63	18	385
	(19/2%)	(12.6%)	(46.9%)	(24.6%)	(48.0%)	(56.8%)	(26.1%)	(31.0%)
Total	271	262	271	134	123	111	69	1241
Other	94	43	124	73	30	58	43

**Table 3.** Behaviors within problem layer.

Behaviors	Low vision	Braille	Total
**Anticipated**			
Point to starting point	8 (28.6%)	**11 (15.1%)**	19 (18.8%)
Point to end point	6 (21.4%)	**13 (17.8%)**	19 (18.8%)
Discuss starting point	2 (7.1%)	**8 (11.0%)**	10 (9.9%)
Discuss end point	9 (32.1%)	**14 (19.2%)**	23 (22.8%)
**Non anticipated**	3 (10.7%)	**27 (37.0%)**	30 (29.7%)
**Total**	28	**73**	101

**Table 4.** Behaviors within design layer.

Behaviors	Low vision	Braille	Total
**Anticipated**			
Point to route	3 (2.1%)	**19 (7.9%)**	22 (5.7%)
Following route	13 (8.9%)	**22 (9.1%)**	35 (9.0%)
Describing route	89 (60.1%)	**89 (36.8%)**	178 (45.9%)
Counting steps route	12 (8.2%)	**7 (2.9%)**	19 (4.9%)
**Non anticipated**	29 (19.9%)	**105 (43.4%)**	134 (34.5%)
**Total**	146	**242**	388

**Table 5.** Behaviors within code layer.

Behaviors	Low vision	Braille	Total
**Bee-bot**			
**Anticipated**			
Make program	0	**8 (2.9%)**	8 (2.6%)
Follow program	1 (3.2%)	**15 (5.5%)**	16 (5.2%)
Press one button	22 (71.0%)	**149 (54.2%)**	171 (55.9%)
Press multiple buttons	0	**64 (23.3%)**	64 (20.9%)
Erase steps	7 (22.6%)	**38 (13.8%)**	45 (14.7%)
**Non anticipated**	1 (3.2%)	**1 (.4%)**	2 (.7%)
**Total**	31	**275**	306
**Blue-bot**			
**Anticipated**			
Make program	1 (.8%)	**4 (3.0%)**	5 (1.9%)
Follow program	2 (1.6%)	**4 (3.0%)**	6 (2.3%)
Take card	49 (39.8%)	**51 (38.1%)**	100 (38.9%)
Put card in reader	44 (35.8%)	**47 (35.1%)**	91 (35.4%)
Take card out reader	12 (9.8%)	**8 (6.1%)**	20 (7.8%)
Change order cards	2 (1.6%)	**2 (1.5%)**	4 (1.6%)
**Non anticipated**	13 (10.6%)	**18 (13.4%)**	31 (12.1%)
**Total**	123	**134**	257

**Table 6.** Behaviors within output layer.

Behaviors	Low vision	Braille	Total
**Anticipated**			
Execute program	14 (18.4%)	**109 (31.2%)**	123 (28.9%)
Follow bot	45 (59.2%)	**165 (47.3%)**	210 (49.4%)
Relate outcome	7 (9.2%)	**40 (11.5%)**	47 (11.1%)
Predict outcome	6 (7.9%)	**27 (7.7%)**	33 (7.8%)
**Non anticipated**	4 (5.3%)	**8 (2.3%)**	12 (2.8%)
**Total**	76	**349**	425

The results consist of two main parts. The first part (3.1) focuses on the abstraction *layers*: their occurrence and the patterns of switching between the layers. This section starts with a display of the occurrence of the layers for each pair within an overall table, which enables a descriptive overview of the frequency of occurrence within and across the pairs. The experience of each pair of switching through the layers while working on the assignment is captured through a description of the pair and an accompanying graph. The graphs should be viewed as qualitative illustrations of the patterns of switching through the layers. The second part of the results (3.2) focuses on the *behaviors* within the layers. This section contains a detailed assessment of the behaviors as they occur in the layers, by reporting and describing the anticipated and non-anticipated behaviors. The focus is more across than within the pairs here, but specific behaviors continue to be ascribed to specific pairs.

## 3. Results

### 3.1. Abstraction layers

**Figure 1.**
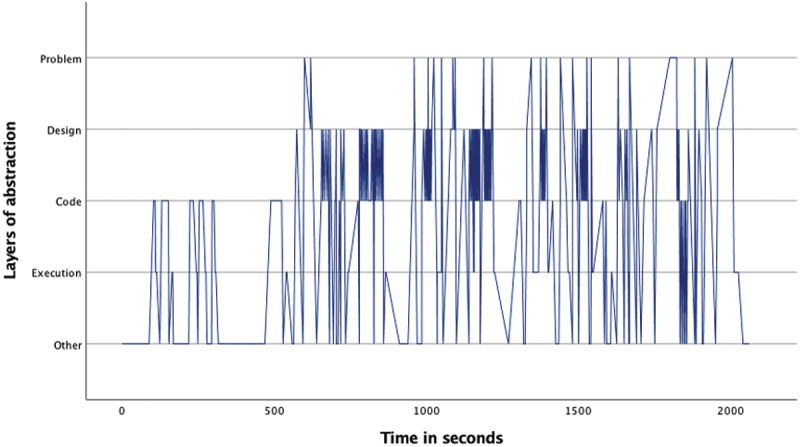
Session 1 pattern of layers.

**Figure 2.**
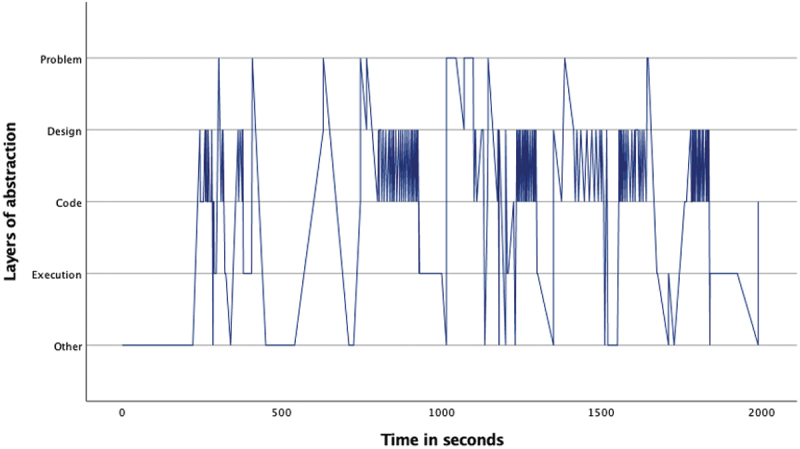
Session 2 pattern of layers.

**Figure 3.**
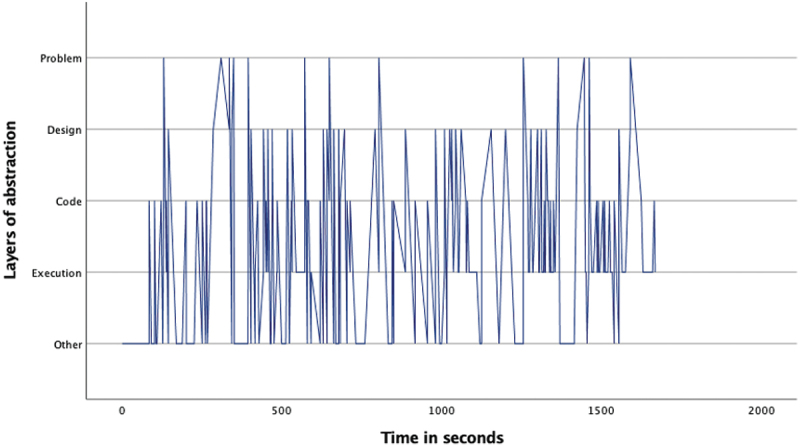
Session 3 pattern of layers.

**Figure 4.**
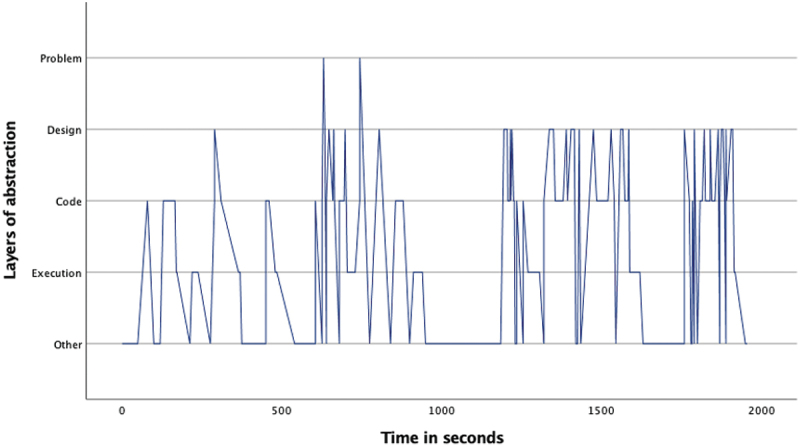
Session 4 pattern of layers.

**Figure 5.**
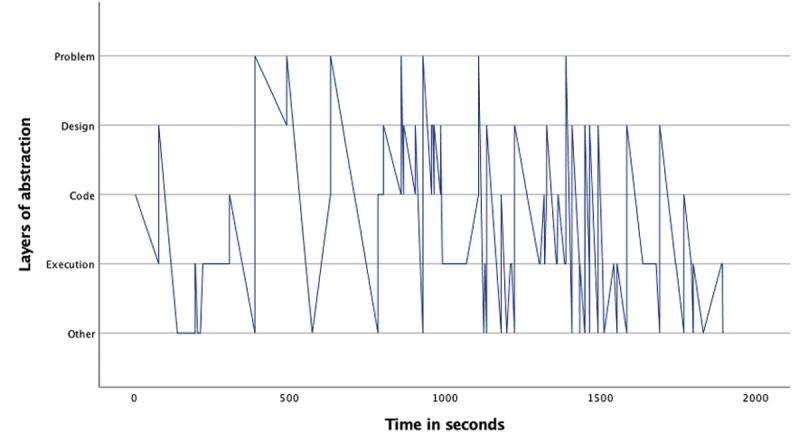
Session 5 pattern of layers.

**Figure 6.**
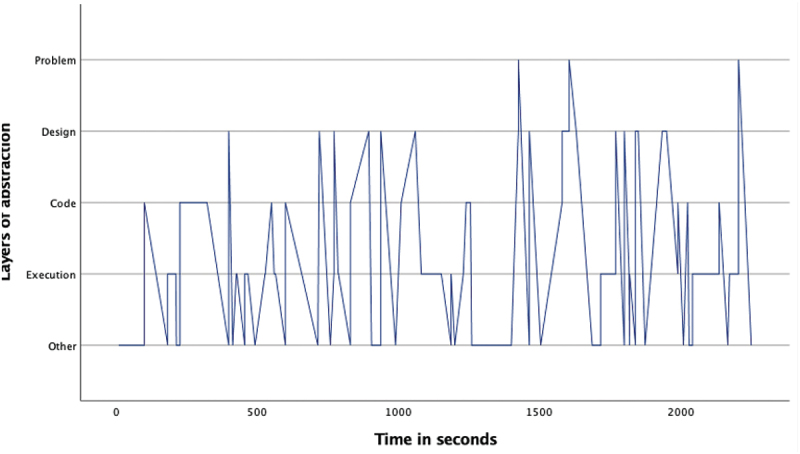
Session 6 pattern of layers.

**Figure 7.**
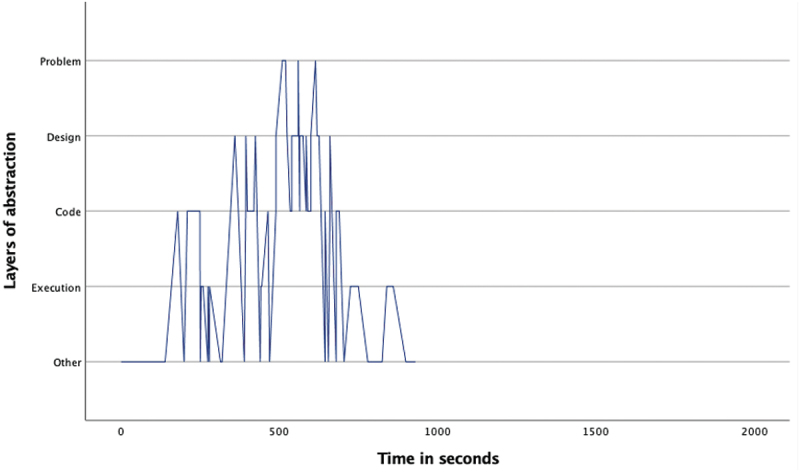
Session 7 pattern of layers.

The overview of the occurrence of the four layers (Table 2) as well as the pattern analysis (Figures 1 to 7) indicates how the children within the seven sessions move and switch through the layers of abstraction while they work on their assignments with the Bee-bot (Sessions 1, 2, 3) and Blue-bot (Sessions 4, 5,6,7). Most sessions lasted around 30 min, session 7 lasted only 15 min. Behavior unrelated to the layers is coded with 0 and displayed in the graphs and included in Table 2 as well. Overall, it can be seen that within all sessions all layers occur. The problem layer occurs least frequently (on average about 7% of the time), as the graphs show it differs per session at which point during the session this usually is. In sessions 1, 2, 3 and 5 the problem layer arises all through the session, whereas in sessions 4, 6, and 7 there are one or two occasions where this layer occurs. The design, code and execution layer usually each take in between 20% and 40% of the behaviors, with some exceptions (for instance the execution layer occurring less often in session 2, and the code layer occurring quite frequently in session 4). In some sessions, the design layer stands out by being less frequently engaged in compared to the code and execution layer (sessions 3 and 5 most clearly) while in session 2 the design layer occurs most often. “Other” behaviors are seen throughout all sessions in between processes related to abstraction. Consistently across sessions, these other behaviors mostly involve the children listening to instructions, generally discussing the material or their collaboration with it, or distractions and actions outside of the material and assignment.

Looking in more detail at the individual graphs, complemented by the accompanying behavior and atmosphere during the different sessions, several observations can be made. A general trend is that sessions 1, 2 and 3 (the Bee-bot sessions) have a denser pattern compared to sessions 4, 5, 6, and 7 (the Blue-bot sessions). Further, especially in sessions 1 and 2 the dense pattern involved several stretches of quickly switching back and forth between coding and designing. Taking a closer look at these sessions, session 1 (Figure 1) concerned two braille learners who often relied on their residual sight by bringing themselves very close to the material. The children preferred to work by themselves and the coding-designing stretches always involved one child programming step by step by pressing the button (code layer) and moving the bot through the environment along to plan the next step (designing). Session 2 consisted of one low vision and one braille learner. The latter did not have any residual sight and relied upon the audio function of the bot and tactile exploration both while programming and while following the bot go on his route, as well as upon quite some verbal and tactile assistance by the tester and the other child. The two boys worked enthusiastically and well together. Whereas Figure 2 shows similar coding-designing stretches as in session 1, in session 2 this always involved both children working together while dividing the tasks, with one child coding and the other child designing. In most of the stretches it was Child1 (low vision) who took on the design and Child2 who coded, only in the third stretch this was the other way around. As the graph indicates, this stretch, which takes places between 1300–1500 seconds within the assignment, is a bit slower paced compared to the other stretches. The tester intensely guided the braille child here in tactilely exploring the maze to think of the next step. In session 3 both children were braille learners (Child1 had some very limited residual sight) primarily using tactile and auditory access, receiving some support by the tester or each other for instance in confirming which button they were to press or in getting oriented. The graph in Figure 3 shows a calmer pattern, which includes the execution layer more frequently in between coding and designing. This reflects the children working on smaller sub-parts of the program which were tested in between. Generally, both boys worked together through the different layers, though Child2 was a bit more active.

The remaining sessions involved the Blue-bot. Session 4 included a braille girl (same as in session 1) and low vision boy, with the graph in Figure 4 indicating a much less dense pattern. The code layer was most frequent here as well, and the problem layer clearly less frequent. There was some, but less frequent and quick, switching back and forth between designing and coding. Because the children did not work well together, similar to session 1 they worked in turn-on designing, coding and running their own program. Session 5 included the same two boys (one braille and one low vision) as session 2. The execution layer was most prevalent here and the graph in Figure 5 is much less dense. This seems to reflect slower steps of coding, where every time the next step was identified the correct card first had to be found. The tasks were not so clearly defined as in their Bee-bot session, coding and designing were both more frequently engaged in by Child 1 who had low vision. In session 6 (Figure 6) as well a similar pair (in this case as in session 3, of two braille learners) worked with the Blue-bot. Their overall experience was much less positive than in their Bee-bot session, they were not very interested in working with the bot anymore and thought it was very limited. The execution layer is again most prevalent, there is however a somewhat slower switching between the four layers, with the problem layer being only included a couple of times later on. Finally, session 7 included a braille girl and low vision girl. This was a relatively short session during which the code layer was most prevalent (Figure 7). The children worked well together and both children are involved through all layers. The braille learner was aware of and asked for what worked for her.

### 3.2. Behaviors within layers

The behaviors the learners displayed within the layers are categorized into anticipated (observed in or referred from previous research) and non-anticipated (first observed in our study). These anticipated and non-anticipated behaviors are indicated in Tables 3 to 6, specified per session as well as by vision type (braille or low vision learner). First, Table 3 provides the behaviors occurring within the problem layer. All anticipated behaviors are engaged in by both low vision and braille learners, though no single behavior is consistently present across all sessions. Discussing the endpoint is overall most frequent within the behaviors occurring within this layer. Further, it can be noticed that relatively more non-anticipated behaviors are engaged in by the braille compared to the low vision learners, taking in almost 40% of all behaviors amongst the braille learners. The inventory of these non-anticipated behaviors showed that they most commonly involved an alternative way to be occupied with the start or ending. This included placing the Bee-bot at the start (multiple times by both children in session 1), discussing the start position/stance of the Bee-bot (once in session 3), coding the final step for the Blue-bot in advance (session 5, by a low vision child), or discussing different routes towards the end. Some additional non-anticipated behaviors that occurred were being generally occupied with the plan or environment, making a drawing of the environment (in session 1) or depicting the goal of the assignment in a physical way. The latter occurred in session 6, and involved Child 1 standing up and physically taking several turns to explain to the other child what kind of turn the bot took (“Look, look, I take a step, turn, step, step, turn, step, turn”).

Second, Table 4 provides the behaviors within the design layer. All anticipated behaviors occurred, and describing the route was by far the most common anticipated behavior, occurring in all sessions. Whereas with the low vision learners this took up over half of their behaviors, in the braille learners this was somewhat less. For the braille learners, similar to the problem layer, non-anticipated behaviors were most common and took up almost half of their behaviors within this layer. The inventory of the non-anticipated behaviors showed that several of these behaviors involved manually moving the bot forward either step by step while programming (Sessions 1, 2, 4) or in one go to plan or check an entire route (Sessions 4, 6 and 7). Though most of this frequently occurring behavior occurred in sessions 1 and 4, in other sessions it was also observed. In session 2 one of the learners (the low vision learner) placed his hand in the route to indicate the position of the bot. Comparable but involving the cards for the Blue-bot was the behavior to hold a card in or next to the route to check its direction. Other behaviors that occurred were drawing a route on paper or working on the environment (which occurred in most sessions).

Third, in Table 5 behaviors within the code layer were inventoried separately for the Bee-bot and Blue-bot since most behaviors within these layers are specific to either bot (involving pressing the buttons on the Bee-bot or picking and placing the cards of the Blue-bot). For the Bee-bot, it can be seen that pressing one button was responsible for around 56% of all behaviors, taking in the majority of behaviors of both low vision and braille learners (though somewhat more in low vision children). Other anticipated behaviors were all observed, though writing code/making the program and pressing multiple buttons not in the low vision children. Non-anticipated behaviors rarely occurred in this layer for the Bee-bot sessions. For the Blue-bot sessions, most frequent were the anticipated behaviors of taking a card and putting the card in the reader, both taking in about 35%–40% of the behaviors and occurring equally frequently in the low vision and braille learners. Other anticipated behaviors occurred but only incidentally. Non-anticipated behaviors did take in about 10% to somewhat more (in the braille learners) of the behaviors, occurring in all Blue-bot sessions. This involved mostly searching a specific card or searching through the cards, correcting the order of the cards in the reader, or interacting with the other child about a card (checking, giving or proposing a card to the other child). Incidentally visual or tactile exploration of a card could be seen as well as (in the Bee-bot sessions) stopping the program or adjusting the bot’s position.

Fourth, concerning the output layer (Table 6), most frequently occurring are following the behavior of the bot (which takes in about 50% of the behaviors within this layer) and executing the program (about 30%). It appears that the low vision children engage in following the bot somewhat more often, and the braille children execute the program. Non-anticipated behaviors occur not regularly within this layer and are only observed 12 times, somewhat more often with the braille learners. Most often this involves stopping a program or moving/adjusting the Blue-bot during the output. Two specific behaviors seen in Session 6 are feeling where the Blue-bot ended up after the program ended, and feeling along with the steps on the Blue-bot while the program is being executed.

## 4. Discussion

In this study, we answered the research question: which patterns and specific behaviors, approached from the layers of abstraction model, do children with visual impairments engage in when working on a programming task with the Bee-bot or Blue-bot? We assessed how nine children (six of whom were blind, three of whom had low vision) move through the four layers of abstraction during programming assignments. Furthermore, we specifically observed which concrete behaviors they employed within each layer. The four layers include the problem layer (the most abstract level where the problem is discussed), the design layer (where a solution is sought), the code layer (where there is direct involvement with the code) and the execution layer (the least abstract level where the code is ran or output is involved) (Faber et al., 2019; Perrenet & Kaasenbrood, 2006; Waite et al., 2018).

### 4.1. Switching between and engaging within the different layers

Overall, across the different sessions the children move through primarily the design, code and output layer, and (to a lesser extent, though present in all sessions) visit the problem layer. The patterns they engage in, including stretches in which they switch back and forth between coding and designing, occasionally visiting the problem layer or the output layer in between, suggest deliberate proceedings involving iterative processes of redesigning and debugging (Lye & Koh, 2014). Whereas in some sessions children focused on coding and thereafter testing the entire program, one pair especially (in sessions 3 and 6) showed an approach of coding and testing sub-parts of the program. Comparing the patterns to the previous study of young sighted learners with the educational bot Cubetto (Faber et al., 2019), it appears our learners show similar overall processes while working on their programming assignment. It can be seen as a valuable addition to the known concrete (relative) usability of the Bee-bot and Blue-bot for visually impaired learners (Jašková & Kaliaková, 2014; Kabátová et al., 2012) that working with these bots also enables to engage in more formal computational processes. The fast connection between input and output has been identified as inviting these processes, for learners overall (Lye & Koh, 2014) and especially for learners with visual impairments (Morrison et al., 2018).

Further, compared to the Bee-bot sessions, the Blue-bot sessions were less dense, showing less behaviors and less fast processes. Coding with the Blue-bot seems obviously a slower process because the action of picking and taking a card takes longer. In addition, the external representation can add to a more conscious and less fast process (Kalas et al., 2018). However, it did not appear coding with the Blue-bot invoked more behaviors in the more abstract layers. It can be considered that for some of the learners the Blue-bot was actually quite difficult, especially after only one Bee-bot session. Consequently, the more sophisticated options of the Blue-bot might not necessarily invite more abstract thinking because of cognitive overload. Cognitive overload is known to occur while learning programming (Mason et al., 2015). Taking into account as well that the tactile versions of the cards of the Blue-bot are in an exploratory phase, this might be especially relevant for learners with visual impairments who generally already need to engage in or learn extra steps (Milne & Ladner, 2018). A most simple tool such as the Bee-bot where all the actions are most clear might free up space to work deliberately.

This observation stresses the importance to continue to improve accessibility also at the level of concrete access to materials such as educational robots. It has been noted before that although tangible materials can be largely accessible for learners with visual impairments, full accessibility is hindered by the inclusion of small visual features (Kabátová et al., 2012; Morrison et al., 2018). From the current context, it can be added that the additional effort or lack of access to part of a tangible material can also actually impact the potential to engage in higher cognitive thinking with the material.

### 4.2. Physical tracing and embodiment in abstraction layers

The exact behaviors low vision and blind learners engage in within the different layers can show how they concretely approach the process of abstraction with these bots. Our learners show a mix of anticipated actions, known from sighted learners (Faber et al., 2019), and spontaneous alternative actions. Furthermore, the extent to which alternative behaviors were used was clearly higher in the problem and design layer (compared to the code and output layer), where they were also more frequently employed by the blind learners (compared to the low vision learners). Taken together, in more abstract layers, more alternative approaches are taken in by blind learners. In interpreting this, it should be taken into account that this observation entails both that alternative actions are required, and that they are possible. The background of the model of the layers of abstraction stipulates that more abstract layers involve moving away from the concrete material, the specific programming language and environment, and move towards an approach to the problem in abstract terms (Perrenet & Kaasenbrood, 2006). In the code and output layer, the blind learners engage regularly in anticipated behaviors, they are actively involved in the assignment by pressing the bot, using the cards and reader, and following the bot. Alternative access for them is inherent in these behaviors themselves: they can see, feel, or hear when they press the buttons as well as when they follow the moving bot. This indicates that at these levels it is mostly about the concrete accessibility of the bot and confirming again the fitting “hybrid” nature (Morrison et al., 2018) of the Bee-bot and Blue-bot. At the more abstract design and problem level however, it becomes more about how the materials facilitate abstract thinking, and the presence of alternative behaviors especially in the blind learners suggests exactly both the need for such behaviors and their possibility while working with the bots.

A further look at the content of the alternative behaviors used in the problem and design layer indicates two ways in which our learners approach abstract concepts or help themselves form a mental model of the assignment. The first approach involves additional direct involvements with the bot. This can be seen both in the problem layer (putting the bot at the start, talking about the positioning of the bot) and, most often and clearly, in the design layer, where the bot is being moved by hand while programming. This way, instead of having the need to mentally represent the steps of the program coded so far as well as the place and orientation of the bot according to these steps within the environment, the bot is used to keep track. Consequently it seems blind learners attempt to keep the connection with the bot while working in the design layer. Previously in the interpretation of sighted learners behaviors, a split was considered within the design layer by making a distinction between “abstract design” (design unrelated to the specific robot or programming language, such as globally describing the route) and “concrete design” (“describe the solution in human language, while also containing elements of the specific robot or programming language”, such as counting squares (Faber et al., 2019)). It could be that the concrete design layer approach is both generally helpful for design-related practices, and supportive for blind learners for whom the direct connection with the bot is beneficial.

A second approach towards abstract concepts shown by our visually impaired learners was physical enactment. This could be seen in the placement of a hand in the route to indicate the position of the bot (in the design layer) and in the interesting case of a blind student physically depicting a turn in order to understand what the type of turn made by the bot entails within the currently planned route (in the problem layer). For a sighted novice learner, it can already be difficult to grasp how the bot makes a “turn in place” without moving a step. Comprehending the size and type of the turn and placing it in the mental image of the route can be even more challenging for a blind learner. Acting out the spatial concept of a turn can be seen as an embodied cognition approach towards spatial thinking and abstract concepts (DeSutter & Stieff, 2017). This approach, which entails that mental processes are mediated by body-based systems which include body shape, movement, and the interaction of the body with the environment, has been explored as an educational strategy within STEM fields and specifically within mathematics (DeSutter & Stieff, 2017; Manches et al., 2020). In the latter field, it has also been explored how embodied cognition can especially help blind learners (Fernandes & Healy, 2010; Healy & Fernandes, 2011). Within the field of computer science education however this approach to facilitate the understanding of abstraction notions has only recently been proposed (Manches et al., 2020) and has not yet been considered for our specific group of learners. Embodied cognition can take several forms, including hand gestures and acting out concepts or representations spatially (Fernandes & Healy, 2010; Healy & Fernandes, 2011; Manches et al., 2020) which could be, as our case example suggests, highly beneficial for learners with visual impairments to conceptualize computing concepts by representing, interpreting, reasoning and communicating about these concepts (Manches et al., 2020). Finally, interestingly this connects to unplugged programming. Unplugged programming activities are often of a physical nature and are moreover known to be activating, engaging and particularly inclusive (Cortina, 2015). Consequently, it can be all the more valuable to further consider how the physical nature of unplugged activities can not only be motivating but also facilitate the learning of abstract computing concepts (Manches et al., 2020) especially for blind learners.

### 4.3. Limitations and future directions

The approach of this qualitative study was to capture our subjects’ experience, within this highly specific and under-explored area of abstract computational concepts in learners with visual impairments. Within our data collection, analysis, and documentation of results, we focused on staying close to the experience of the subjects, in a tailored and detailed manner (Guba, 1981; Krefting, 1991). Consequently, our small and specific sample was fitting within this aim and approach, and generalization was not the primary intent. However, there are some limitations connected to our approach and set-up. First, we included solely learners with visual impairments, as opposed to sighted individuals as well. The direct comparison of the latter set-up would enable a fuller interpretation of young learners’ computational practices and underlying mental modeling and abstract thinking using the Bee-bot and Blue-bot in programming assignments. Given the complexities and diversity within the group of learners with visual impairments, a focused investigation into low vision and blind children can be seen as a suited first step. Relatedly, we do consider transferability of our findings to similar participants and contexts possible. The diversity of our sample should be carefully taken into account here, in terms of both vision and other specificities. Currently, our findings might be most directly applicable to special education settings, where this diversity is always present and adapted to. In other settings, the insights and ideas generated in this study can be further explored.

Second, in the interpretation of our findings, the concrete accessibility of the set of materials of specifically the Blue-bot for blind and low vision learners should be taken into account. Although adapted tactile versions of the Blue-bots cards, designed for learners with visual impairments, were used in our study, these cards are still in a development phase. Consequently, the set of Blue-bot materials is currently not completely (validated) accessible. This could impact how well learners with visual impairments can engage in computational practices with the material, and it emphasises the need to continue to improve accessibility at the concrete level of materials. At the same time, however, how well individuals with visual impairments can work or learn with a not entirely accessible material is also in line with their daily context, where specifically in the case of computers and technology often it is necessary to “work around” accessibility issues (Albusays & Ludi, 2016; van der Meulen et al., 2022). Third, in our data processing we relied upon one coder using a detailed coding scheme that was partially fixed and partially allowed for new observations within fixed categories. This approach fitted our qualitative study set-up, yet there is the risk that personal interpretation of the coder could have impacted the interpretation of the behaviors.

Future studies could focus on continuing to understand learners with visual impairments’ approach to the process of abstraction, also by involving and directly comparing their processes and behaviors with those of sighted learners. Further, in order to support the development of computational concepts such as abstraction in visually impaired learners, supportive teaching strategies and instructions should be developed in connection to insights on approaches by these learners. Specific directions suggested in our findings, such as an embodied cognition approach, could be further explored as potentially helpful within early programming education in general, and especially for learners with visual impairments. A valuable option would be to make the connection with unplugged lessons as well.

## 5. Conclusion

Our findings show that learners with visual impairments, using the Bee-bot and Blue-bot, engage in a formal computational way of working within the process of abstraction, including iterative actions of redesigning and debugging. Further, they engage in these computational practices using a mix of behaviors known from sighted learners as well as, especially the blind learners in the more abstract layers, alternative behaviors. The content of the latter indicates the preference to be physically involved and keep track of the bot and the plan. Moreover, it suggests how embodied cognition in the form of physical enactment can be helpful to grasp an abstract concept and mental representation. Overall, the previous operationalization of the model of the layers of abstraction in sighted learners can be meaningfully applied to low vision and blind learners, when elaborated with specific tactile and physical behaviors. Furthermore, such behaviors can be further established, possibly as part of an embodied cognition approach within inclusive computer science education, in order to encourage teachers to support learners with visual impairments in their conceptualization of abstract notions and mental representations within programming education.
